# Exploring the Bioactive Potential of *Calostoma insigne,* an Endangered Culinary Puffball Mushroom, from Northeastern Thailand

**DOI:** 10.3390/foods13010113

**Published:** 2023-12-28

**Authors:** Worachot Saengha, Thipphiya Karirat, Nathanon Pitisin, Supawadee Plangklang, Luchai Butkhup, Piyachat Udomwong, Nyuk Ling Ma, Ampa Konsue, Pornwipa Chanthaket, Teeraporn Katisart, Vijitra Luang-In

**Affiliations:** 1 Natural Antioxidant Innovation Research Unit, Department of Biotechnology, Faculty of Technology, Mahasarakham University, Maha Sarakham 44150, Thailand; worachot207@gmail.com (W.S.); thipphiya.k@gmail.com (T.K.); ptsmabel@gmail.com (N.P.); pksupawadee.16@gmail.com (S.P.); luchai.b@msu.ac.th (L.B.); 2 International College of Digital Innovation, Chiang Mai University, Chiang Mai 50200, Thailand; piyachat.u@cmu.ac.th; 3 BIOSES Research Interest Group, Faculty of Science and Marine Environment, Universiti Malaysia Terengganu, Kuala Nerus 21030, Terengganu, Malaysia; nyukling@umt.edu.my; 4 Thai Traditional Medicinal Research Unit, Division of Applied Thai Traditional Medicine, Faculty of Medicine, Mahasarakham University, Maha Sarakham 44000, Thailand; ampa.k@msu.ac.th; 5 Ban Khwao Forest Temple, Maha Sarakham 44000, Thailand; aor.chawala1@gmail.com; 6 Department of Biology, Faculty of Science, Mahasarakham University, Maha Sarakham 44150, Thailand; tkatisart@gmail.com

**Keywords:** antidiabetic activity, anticancer, antioxidant, antimicrobial activity, polysaccharide, HT-29 cells

## Abstract

*Calostoma insigne* puffball mushrooms are only found in forests with rich biodiversity in very few countries including Thailand, and their biofunctions remain largely unexplored. This study used the agar disk diffusion assay, the anti-glucosidase assay, and the 3, 4, 5-dimethylthiazol-2-yl-2-5-diphenyltetrazolium bromide (MTT) assay to evaluate the bioactive potential of these endangered puffball mushrooms. Internal transcribed spacer (ITS) gene analysis identified *C. insigne*, a puffball mushroom with green, globose, and spiny spores. Fourier-transform infrared spectroscopy (FTIR) analysis confirmed the polysaccharide structure while scanning electron microscopy (SEM) revealed a fiber-like network. The ethanolic gelatinous fruiting body extract exhibited 1,1-diphenyl-2-picrylhydrazyl (DPPH)-scavenging capacity (57.96%), a ferric ion-reducing antioxidant power (FRAP) value of 1.73 mg FeSO_4_/g, and α-glucosidase inhibition (73.18%). *C. insigne* cytotoxicity was effective towards HT-29 colon cancer cells using the MTT assay (IC_50_ of 770.6 µg/mL at 72 h) and also showed antiproliferative capacity (IC_50_ of 297.1 µg/mL). This puffball mushroom stimulated apoptotic genes and proteins (caspase-3, Bax, and p21) via an intrinsic apoptotic pathway in HT-29 cells. In the laboratory, the medium formula consisting of 20% potato, 2% sucrose, and 0.2% peptone was optimal to increase fungal mycelial biomass (2.74 g DW/100 mL), with propagation at pH 5.0 and 30 °C. Puffball mushrooms are consumed as local foods and also confer several potential health benefits, making them worthy of conservation for sustainable utilization.

## 1. Introduction

Gasteroid fungi, characterized by their distinctive method of spore distribution and appealing fruiting structures, exemplify an intriguing facet of fungal biodiversity. Among gasteroids, the genus *Calostoma* encompasses a unique variety of mushrooms usually referred to as “puffballs” or “eyeballs” [1]. Fungi in the *Calostoma* genus, family Sclerodermataceae and order Boletales [1], have long fascinated scientists due to their ecological functions and evolutionary adaptations. *Calostoma* sp. are distributed throughout Australasia, Asia, and Eastern North America [2,3]. The exoperidium of puffball mushrooms has exquisite ornamentation, with ostioles resembling human lips, and thus, they are also called “pretty mouths” (World Heritage Encyclopedia, 2021) [4].

Recently, interest in the therapeutic capabilities of mushrooms has been driven by the identification of bioactive substances with diverse health-enhancing properties including anticancer, antidiabetic, antioxidant, antimicrobial, antihypertensive, anti-inflammatory, immunomodulatory, neuroprotective, and cholesterol-lowering in multiple types of fungi [5,6,7,8,9,10,11]. *Calostoma* mushrooms occur in several countries but the literature confirming their edibility is limited to Malaysian *C.* cf. *fuscum*, Thai *C. junghuhnia*, and Mexican *C. cinnabarinum* [12,13,14], while their human health benefits remain largely unexplored.

*Calostoma insigne* is found in Borneo, Java, Sumatra, New Guinea, Malaysia, Thailand, Papua New Guinea, and the Philippines [15], thriving in Southeast Asian wet tropical rainforests. This mushroom grows on soil and rotting leaves on hills during the wet season. Isotopic labeling, molecular, and morphological studies have shown that the species is ectomycorrhizal [15] and symbiotically bonds with Dipterocapceae trees [15] that dominate Southeast Asian neotropical forests [16]. With a loss of over 50% of forest area predicted from 2000 to 2050 due to global warming, the population of this species is expected to decline [17]. *C. insigne* currently falls into the Red List criterion of A4c, meaning that it is endangered according to the IUCN Red List of Threatened Species (2019) [15]. Thus, this species should be conserved and studied for propagation in laboratories and farms as a local food resource.

In Thailand, *C. insigne* is nicknamed “Hed-Ta-Lo”, which means “Big Eye Mushroom”. It is only found in a few provinces in Northeastern Thailand during the rainy season (August–October), growing in forests with rich biodiversity. The local people harvest this mushroom and eat its exoperidium (gelatinous layers) and gleba (spore mass) raw, believing that it displays antimicrobial activity. Thai Forest Monks believe that consuming this mushroom alleviates inflammatory bowel illness, human gut diseases, and diabetes because the cooling and softening properties of the gelatinous layers act as the water element to quench the body’s fire element. However, this traditional wisdom for the potential health benefits of *C. insigne* has not been scientifically proven. Thus, this study investigated the antioxidant, antimicrobial, anti-glucosidase, and anticancer activities of *C. insigne,* an endangered culinary puffball mushroom, for the first time. The optimal laboratory medium for *C. insigne* and mycelial biomass growth was also evaluated to boost conservation and achieve the Sustainable Development Goals (SDG).

## 2. Materials and Methods

### 2.1. Mushroom Extract Preparation

During the rainy season (9 September 2021), *C. insigne* puffball mushrooms were collected at the immature fruiting body (reproductive) stage from Ban Lao Khae Local Forest, Ban Lao Sua Kok, Lao Sua Kok Subdistrict, Trakan Phuet Phon District, Ubon Ratchathani Province, Thailand, and taxonomically identified at the Biotechnology Department, Mahasarakham University, Thailand, under voucher specimen number of BIOTMSU F01. The mushrooms were rinsed and cleansed with distilled water. Only the gelatinous tissues of the immature fruiting bodies (without gleba mass or spores) were used as one sample batch for extraction. These tissues were oven-dried at 40 °C and then delicately pulverized. The gelatinous fruiting bodies were subjected to extraction in 95% ethanol using a maceration method at room temperature for 24 h. After drying using a rotary evaporator, the desiccated substance obtained was frozen at −20 °C for further analysis.

### 2.2. Mycelial Growth of the Fungal Isolate

After rinsing and cleansing with autoclaved distilled water, the mushrooms were cut open through the middle with a sterilized knife. The white powdery dust-like spores inside the fruiting body were placed on potato dextrose agar (PDA) in triplicate and incubated at 30 °C for 8 days with the mycelial diameter measured daily.

### 2.3. Microscopic Observation of Spores and Gelatinous Tissues

The shape and size of fungal spores in the gleba of the fruiting body were determined under a light microscope. Spore preparation without any treatment for scanning electron microscopy (SEM) analysis was conducted as previously described [18]. The morphology and characteristics of spores, capillitium, fresh gelatinous tissues, and freeze-dried gelatinous tissue powder were observed using a SEM Leo/1450 (Carl Zeiss, Oberkochen, Germany) at 15 kV.

### 2.4. Molecular Identification of the Fungal Isolate and Phylogenetic Tree Construction

The pure fungal isolate was sub-cultured in a 250 mL Erlenmeyer flask with 100 mL potato dextrose broth (PDB) (Oxoid, Basingstoke, UK) for 7 days. A fungal mycelial mass was filtered from the broth using sterile No. 5 Whatman filter paper. The mycelial mass was crushed in a porcelain mortar and extracted for genomic DNA using UniversAll tissue extraction buffer (Diagnocine, Totowa, NJ, USA) as per the manufacturer’s instructions. The fungal isolate was identified using ITS5F (5’-GGAAGTAAAAGTCGTAACAAGG-3’) and ITS4R (5’-TCCTCCGCTTATTGATATGC-3’) primers. For polymerase chain reaction (PCR), a 20 µL reaction mixture was prepared with 1 µL DNA, 0.2 µL DNA polymerase, 0.5 µL forward and reverse primers, 1 µL dNTPs, and sterile ddH_2_O. The PCR conditions included initial denaturation at 94 °C for 3 min, 35 cycles of denaturation for 40 s, annealing at 55 °C for 40 s, extension at 72 °C for 1 min, and a final extension for 7 min. After amplification, the PCR products were gel electrophoresed on 1% agarose gel (1 g in 100 mL Tris solution) stained with Sybr Safe dye. DNA sequencing was performed on the ITS gene PCR product and BLAST search in NCBI was conducted to find the closest relative species, with MUSCLE for multiple sequence alignments [19]. Maximum likelihood [20] based on the Tamura-Nei model [21] was used to create the phylogenetic tree, with the proportion of bootstrap test trees with associated taxa (1000 repetitions) next to the branches [22]. Each site was 0.2 substitutions away from the horizontal bar. MEGA11 was used to create the phylogenetic tree and perform evolutionary studies [23,24].

### 2.5. Optimal Culture Medium for Mycelial Growth

Four media broth formulae were tested for optimal mycelial growth as T1 = PDB (Control), T2 = 20% potato + 2% sucrose, T3 = 20% potato + 2% sucrose + 0.2% peptone, and T4 = 20% potato + 2% sucrose + 0.5% malt extract. Potato slices (200 g) were cooked in 1000 mL distilled water for 15 min. The boiling solutions were cheesecloth-filtered, and the filtrates were combined with sucrose or nitrogen following the above formulae and adjusted to 1000 mL with distilled water at pH 5.0. All culture media were autoclaved at 121 °C for 15 min. A cork borer was used to cut three 4 mm mycelial discs from an inoculum culture (7 days of growth) on PDA and transfer them to 250 mL flasks with different broth formulae (100 mL). The cultures were cultivated for 5 days at 30 °C and 150 rpm. Mycelial fresh and dry weights were measured. To achieve a constant dry weight, mycelial pellets from 100 mL of each broth were filtered using Whatman No. 1 paper and dried at 40 °C. The dry weight of the mycelial pellets in triplicate was obtained using a digital balance. The dry mycelia were then extracted with ethanol in the same way as mentioned in the gelatinous tissue ethanolic extraction. The mycelial extracts were tested for antioxidant activity and bioactive contents as follows.

### 2.6. Fourier-Transform Infrared Spectroscopy (FTIR) Analysis

The chemical bond characteristics of desiccated gelatinous tissue and mycelial extract (1 mg) ground in KBr particles (20 mg) in a 1:20 *w*/*w* ratio were recorded using a Spectrum GX (PerkinElmer Inc., Waltham, MA, USA) between 500 and 4000 cm^−1^. Dextran was used for comparison.

### 2.7. Antioxidant Activity and Bioactive Contents

The antioxidant capacity of the gelatinous fruiting body and mycelial extracts (20 mg/mL) were tested in triplicate by the following assays.

#### 2.7.1. DPPH Radical-Scavenging Activity

The ability of the matched extracts to donate electrons or hydrogen atoms was assessed by bleaching a purple-colored methanol solution of the stable DPPH free radical, following the steps of a previous report [25]. Twenty microliters of 20 mg/mL extract were mixed with 180 μL of 10 mM DPPH• (dissolved in ethanol) (Sigma-Aldrich, St. Louis, MO, USA). After 30 min, A_515nm_ was measured using a M965+ microplate reader (Metertech, Taipei, Taiwan), and DPPH• scavenging ability (%) = (1 − A_sample_/A_control_) × 100 (A_control_ = absorbance without sample; A_sample_ = absorbance with sample). A standard curve of Trolox was also created to calculate the DPPH-scavenging activity as mg Trolox/g extract in triplicate.

#### 2.7.2. Ferric-Reducing Antioxidant Power (FRAP) Assay

Reductants (antioxidants) decrease Fe^3+^ to the blue Fe^2+^-tripyridyltriazine complex which can be recorded at 593 nm absorbance using a M965+ microplate reader (Metertech, Taipei, Taiwan), after a 30 min incubation. The FRAP assay was performed as per an earlier report [25] by mixing 20 μL of extract (20 mg/mL) with 180 μL of FRAP reagent (20 mM FeCl_3_, 10 mM 2,4,6-Tri (2-pyridyl) s-triazine, 0.3 M acetate buffer, pH 3.6). A standard curve with known FeSO_4_ amounts was used to determine the results in mg FeSO_4_/g extract in triplicate.

#### 2.7.3. Determination of Total Phenolic and Flavonoid Contents

Folin–Ciocalteu reagent was used to measure the total phenolic content (TPC) as per the previous protocol [26]. The mixture consisted of 100 μL of 10% Folin–Ciocalteu solution, 20 μL of extract (20 mg/mL), and 80 μL of 7.35% sodium carbonate. A_725nm_ was recorded using a M965+ microplate reader (Metertech, Taipei, Taiwan) after 30 min at room temperature. Results were reported as mg gallic acid equivalent (GAE)/g of extract. The colorimetric approach [26] was used to measure the total flavonoid content (TFC) by mixing the extract (20 mg/mL) with deionized water (60 μL), 10% aluminum trichloride (10 μL), and 5% sodium nitrate (10 μL). A_420nm_ was measured after 30 min of reaction with 100 μL of 1 M NaOH, with results shown as mg rutin equivalent (RE)/g of extract in triplicate.

### 2.8. Antimicrobial Activity

Three bacterial pathogens were tested. *Staphylococcus aureus* TISTR 517 (ATCC 25923) (GenBank accession no. OP522324.1) and *Escherichia coli* TISTR 527 (ATCC 11775) (GenBank accession no. X80725.1) were obtained from Thailand Institute of Scientific and Technological Research (TISTR), while *Streptococcus agalactiae* EW1 was sourced from diseased Nile tilapia in the northeastern region [27]. Luria–Bertani (LB) broth (HiMedia, Maharashtra, India) was used to grow all bacteria to 10^8^ CFU/mL after 24 h at 37 °C. Each 4 mm paper disc was pipetted with 20 μL of sterilized extract (20 mg/mL), placed on LB agar plates inoculated with 100 μL of bacterial suspension, and cultivated at 37 °C for 48 h. The inhibitory zone (mm diameter) as antimicrobial activity was measured in triplicate. Ten μg/mL penicillin (Sigma-Aldrich, St. Louis, MO, USA) was used as a positive control.

### 2.9. α-Glucosidase Inhibition Assay

The inhibition of α-glucosidase was measured following the method of Wongsa et al. (2012) [28] in triplicate, with some adjustments. Each extract (20 μL of 20 mg/mL stock) was combined with 0.1 mL of 0.1 M potassium phosphate buffer (pH 6.9) containing 1 U/mL α-glucosidase solution. After thirty minutes, 50 μL of 5 mM *p*-nitrophenyl-α-D-glucopyranoside solution in 0.1 M potassium phosphate buffer (pH 6.9) was added to the mixture at 37 °C for 5 min. The absorbance at 405 nm reading before and after incubation was compared to the control, which contained 50 μL of buffer solution instead of the extract. α-Glucosidase inhibition (%) = [(ΔA_o_ − ΔA_e_)/ΔA_o_] × 100, where ΔA_o_ is absorbance without sample, and ΔA_e_ is absorbance with sample.

### 2.10. Cytotoxic Activity

Cytotoxic activity of the gelatinous tissue of the fruiting body extract towards five types of cancer cells, i.e., HepG2 (liver), MCF-7 (breast), HeLa (cervix), A549 (lung), and HT-29 (colon), was assessed using the MTT assay. The overnight growth of cancer cells (5 × 10^3^ cells/well) in 96-well plates was prepared. Extracts (0–800 µg/mL diluted in medium) were tested on cancer cells for 24 h in triplicate. MTT reagent was used to replace the medium, and the cells were left to react for 4 h. Afterward, 200 µL of dimethylsulfoxide (DMSO) was used to dissolve the formazan crystals, and A_590nm_ was read. All chemicals were sourced from Sigma-Aldrich, St. Louis, MO, USA. IC_50_ and cytotoxicity (%) of the extracts in triplicate was measured. % Cytotoxicity = [(A_o_ − A_e_)/A_o_] × 100, where A_o_ = absorbance without sample and A_e_ *=* absorbance with sample.

### 2.11. Clonogenic Assay

Anti-colony formation activity of the gelatinous fruiting body extract was measured [25]. The 6-well plates each received 500 cells of HT-29. Following 24 h incubation, the cells were subjected to the gelatinous fruiting body extract (0, 100, 200, 400, 500, 800 µg/mL) in a new medium at 37 °C with 5% CO_2_. The cells were then rinsed twice with phosphate-buffered saline (PBS) and cultured for another 14 days in new DMEM medium, which was refreshed every 3 days. The cells were then rinsed with PBS and fixed with methanol for 1 h. After staining with 0.5% Coomassie brilliant blue g-250 in methanol for 30 min, the colonies were rinsed with water and photographed. Colony formation (%) and IC_50_ in triplicate were calculated.

### 2.12. Determination of Caspase-3 Activity

A commercial kit (Abcam, Cambridge, UK) was used to evaluate caspase-3-dependent apoptosis. After a 24 h treatment of HT-29 cells (1.5 × 10^5^/well) with gelatinous fruiting body extract (0, 100, 200, 400, 600 µg/mL), the cells were detached and washed twice with cold PBS (1500× *g*, 4 °C, 5 min). RIPA lysis buffer was used to lyse the cells. Bicinchoninic acid reagent was used to measure cellular protein concentrations. The cell lysate was combined with 50 μM DEVD-AFC substrate in buffer, incubated at 37 °C for 90 min in the dark, and then read in a fluorometer with 400 nm excitation and 505 nm emission filters. The fold change in caspase-3 activity in triplicate compared to untreated cells (control) was recorded.

### 2.13. Intracellular Reactive Oxygen Species (ROS) Detection

The reagent 5-(and-6)-carboxy-2′,7′-dichlorodihydrofluorescein diacetate (DCFDA) (Thermo Fisher Scientific, Rockford, IL, USA) was used to assess intracellular hydroxyl and peroxyl radical production. The experiment involved seeding HT-29 cells (2 × 10^4^ cells/well) into black 96-well plates followed by incubation for 24 h. The gelatinous fruiting body extract (0, 100, 200, 400, 600 µg/mL) with DCFDA (20 µM) was applied to cells in a serum-free medium and incubated at 37 °C for 2 h. When intracellular ROS levels rose, DCFDA became DCF, a green, fluorescent molecule, which was measured at 485 nm excitation and 535 nm emission using a fluorescence microplate reader and captured with an EVOS M5000 Imaging System fluorescence microscope. In the fluorescent cell imaging, DNA was stained with Hoechst 33342 (3 µM) solution. ROS generation (fold change) was compared to the untreated control. All measurements were conducted in triplicate.

### 2.14. Intracellular Mitochondrial Membrane Potential (ΔΨm) Assay (JC-1)

A mitochondria-specific dual fluorescence probe was used to measure the mitochondrial membrane potential. After overnight seeding, HT-29 cells (2 × 10^4^ cells/well) were treated with gelatinous fruiting body extract (0, 100, 200, 400, 600 µg/mL) for 24 h in black 96-well plates to which JC-1 working solution (4 μM) was the added and incubated at 37 °C for 30 min. The cells were washed twice with PBS, replaced with new culture media, and incubated in 100 µL/well of JC-1 buffer at 37 °C for 30 min. The fluorescent intensity was measured at 485 nm excitation and 535 nm emission using a fluorescence microplate reader and an EVOS M5000 Imaging System confocal fluorescence microscope. Red/green fluorescence intensity ratios were measured to assess mitochondrial membrane potentials. Red fluorescence (535 nm laser stimulation) showed JC-1 aggregates in polarized mitochondria and normal physiological conditions. Green fluorescence signals (485 nm laser excitation) showed JC-1 monomers dominated depolarized mitochondria, causing mitochondrial membrane potential loss, malfunction, and apoptosis. Red/green fluorescence intensity ratio decreased, indicating mitochondrial membrane depolarization. DNA was stained with Hoechst 33342 (1 µg/mL) in fluorescent cell imaging. Triplicate measurements were conducted.

### 2.15. Annexin V-FITC/PI Staining

Early/late apoptosis and necrotic cells were identified using Annexin V-FITC and PI labeling. After incubating HT-29 cells (2 × 10^4^ cells/mL) in a chamber slide for 24 h, the cells were treated with gelatinous fruiting body extract for another 24 h. The medium was taken from the cell culture slide to monitor apoptotic and necrotic cells by adding 1 µg/mL Annexin V and 1 µg/mL PI to the binding buffer (10 mM HEPES, 140 mM NaCl, 2.5 mM CaCl_2_ pH 7.4) and incubating for 15 min. The DNA was stained with Hoechst 33342 (1 µg/mL). A fluorescent microscopy EVOS M5000 Imaging System was used to capture stained cells. Triplicate measurements were conducted.

### 2.16. Real-Time PCR Analysis

After 24 h, HT-29 (2 × 10^5^ cells/well) grown in 6-well plates at 37 °C were subjected to the gelatinous fruiting body extract (0, 200, 400, 600 µg/mL) in fresh media. RNA, isolated with TRIzol™ reagent (Invitrogen, UK) (1 μg/sample), was transcribed to cDNA using iScript™ Reverse Transcription Supermix (Bio-Rad, Hercules, CA, USA). Quantitative real-time PCR to evaluate gene expressions was conducted using a CFX Duet Real-Time PCR System and iTaq Universal SYBR Green Supermix (Bio-Rad, Hercules, CA, USA). Table S1 lists the primers for *Bax*, *Bcl2*, *caspase-3*, and *p21*. The PCR conditions were as follows: 5 min at 94 °C (denaturation) and 10 s at 60 °C (annealing and extension) for 40 cycles. GAPDH was used to standardize the expression, with the 2^−ΔΔCT^ method used to analyze CT values statistically. Triplicate measurements were conducted.

### 2.17. Protein Extraction and Western Blot Analysis

The HT-29 cancer cells were plated at 2.5 × 10^5^ cells/well in 6-well plates for 24 h before treatment with gelatinous fruiting body extract (0, 400, 600 µg/mL). After collection, living cells were washed with ice-cold PBS and lysed in RIPA buffer (50 mM Tris-Cl pH 7.4, 150 mM NaCl, 1% NP-40, 0.5% sodium deoxycholate, 0.1% SDS) with protease and phosphatase inhibitor cocktail (Roche, Penzberg, Germany) for 30 min on ice and centrifuged at 14,000× *g*. The supernatant was collected, and a BCA protein kit test (Thermo Fisher Scientific, Rockford, IL, USA) was used to assess protein content. Protein samples (20 µg) were then heated at 98 °C for 10 min in a loading buffer with 100 mM dithiothreitol (DTT). Protein samples were resolved with SDS-PAGE on 12% polyacrylamide gel electrophoresis and transferred to a PVDF membrane at 90 V for 1 h. The membranes were blocked for 1 h with 5% bovine serum albumin in Tris-buffered saline containing 0.1% Tween-20 (TBST) and incubated overnight with rabbit polyclonal anti-human Bax, caspase-3, p21, and GAPDH (internal control) antibodies at 4 °C. After three TBST washes, the membranes were incubated with 1:5000 secondary Ab coupled with horseradish peroxidase at room temperature for 1 h. Chemiluminescent detection was performed using Amersham ECL TM Prime after washing the membrane in TBST. The ChemiDoc Imaging Systems (Bio-Rad, Hercules, CA, USA) was used to monitor protein band densities. Triplicate measurements were conducted.

### 2.18. Statistical Analysis

The data were analyzed using ANOVA and presented as mean ± SD of triplicate values in all analyses. Significant differences were determined with Duncan’s new multiple range test using the SPSS package Demo version (IBM, Armonk, NY, USA). Statistical significance was set at *p* < 0.05.

## 3. Results

### 3.1. Characterization of the Fungal Isolate and Spores

**Figure 1:**
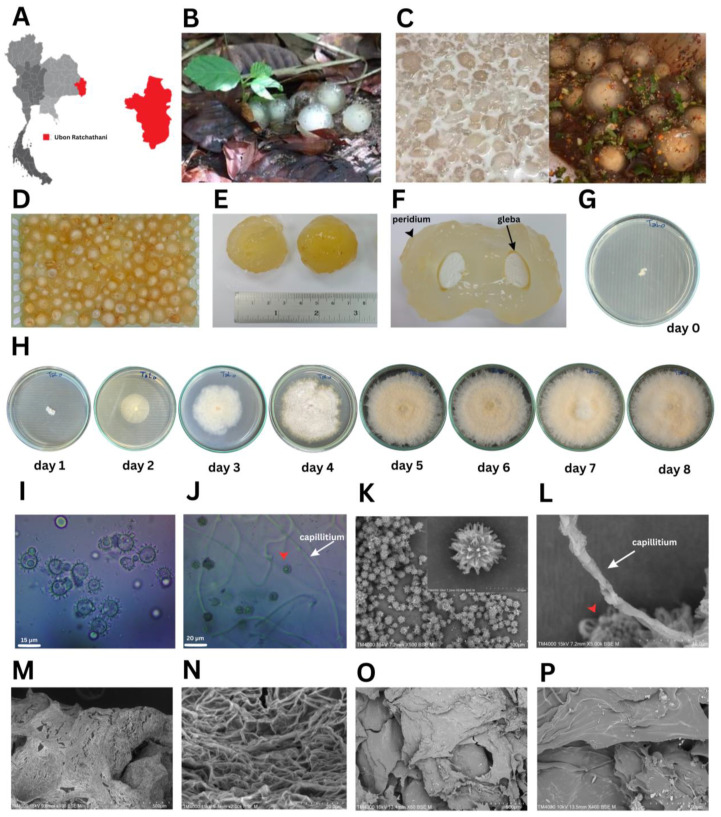
Puffball mushroom morphological characteristics: (**A**) map of Thailand showing where the puffball mushrooms were collected in Ubon Ratchathani Province (red), (**B**) puffball mushrooms on the ground under foliage, (**C**) dessert in coconut milk (**left**) and Koi Hed Ta Lo in spicy condiment (**right**), (**D**) puffball mushrooms after washing, (**E**) diameter of puffball mushrooms, (**F**) white powdery spores “gleba” (arrow) and the exoperidium (arrowhead), (**G**) spores placed on PDA at day 0, (**H**) mycelial growth on PDA at day 1–8, (**I**) light microscopic morphology of fungal green spiny spores at 400×, (**J**) microscopic morphology of spores (red arrowhead), and hyaline gelatinous hyphae (black arrow) at 1000×, (**K**) spores under SEM at 500× and 5000×, (**L**) capillitial thread at 5000×, (**M**) fresh hyaline gelatinous hyphae at 100×, (**N**) enlarged image of M at 2000×, (**O**) freeze-dried powder of gelatinous tissue of the fruiting body at 60×, and (**P**) enlarged image of O at 400×.

Puffball mushrooms at the immature fruiting body stage were collected from Ubon Ratchathani Province, Thailand (Figure 1A). These morphologically unique basidiomycetes fruits grow with minimal nutrients on sandy soil with decomposing deciduous tropical forest leaves (Figure 1B). The main species of Dipterocarp trees are Shorea and Hopea [29]. The young mushrooms are commonly eaten raw by the local people as a Thai dessert in coconut milk (Figure 1C, left panel) and as ‘‘Koi-Hed-Ta-Lo’’, a spicy condiment, mixed with chili powder, roasted rice, and fish sauce (Figure 1C, right panel). The shape is like an egg or human eyeball with clear brightly colored thick gelatinized tissue (Figure 1D) that looks like the flesh of a lychee fruit. Mushroom diameters commonly range from 3.0–4.0 cm (Figure 1E), although big balls of 6.00 cm in diameter can be found. Once cut open, the white powdery spores inside the mushrooms called “gleba” were revealed (Figure 1F). These white powdery spores (2.05 ± 0.07 mm in diameter) were placed on PDA at 30 °C at day 0 (Figure 1G). The mycelial diameter was measured daily for 8 days (Figure 1H). Results showed that mycelial growth started as a white colony on day 1 (4.10 ± 0.14 mm), and then grew on day 2 (24.25 ± 0.35 mm), day 3 (35.25 ± 0.35 mm), day 4 (50.50 ± 0.71 mm), day 5 (80.75 ± 0.35 mm), day 6 (83.50 ± 0.71 mm), and days 7 and 8 (9.00 ± 0.00 mm), with the mycelia covering a full PDA plate (Figure 1H). The microscopic morphology showed globose green basidiospores with coarse spiny ornamentation (13.5–17.1 µm in diameter) and capillitial threads (1.51–1.88 µm in width) (Figure 1I,J) under a light microscope. In the SEM observation, basidiospores were adorned with projecting spines, and aseptate capillitial threads were seen (Figure 1K,L). These projecting spines or star-shaped forms connect the immature basidiospores to their basidia and mycelial network in the fertile area (the gleba). The fresh gelatinous tissues of the fruiting body showed numerous interwoven cord-like fibers (Figure 1M,N), whilst its freeze-dried powder showed a compact and smooth texture (Figure 1O,P). The basidiocarp structures of *Calostoma* sp. are more refined than those of other gasteroid fungi, with pitted-spore reticulations being particularly intricate. By interlacing reticulated basidiospores with nurse cells and capillitial hyphae that are densely scaled, spores are shielded from being carried away simultaneously [30,31]. The spore-bearing portion of the Calostoma, or vegetative portion, is composed of numerous cord-like fibers that are tenacious, translucent, and jelly-like. These fibers form a dense network by branching and anastomosing.

### 3.2. Phylogenetic Analysis

**Figure 2:**
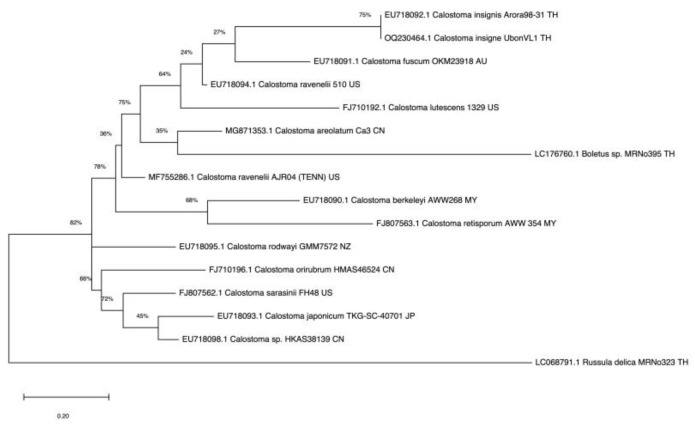
Phylogram for *Calostoma* generated from maximum likelihood analysis of sixteen ITS sequences.

Sixteen ITS sequences from mushrooms of *Calostoma, Boletus* (relative), and *Russula* (outgroup) genera were aligned. The fungal isolate from the puffball mushroom collected in Ubon Ratchathani Province, Thailand, was identified as *C. insigne* (GenBank accession no. OQ230464.1); it was evolutionarily closest to *C. insignis* Arora98-31 (EU718092.1) found in Thailand [32], with 99% sequence similarity based on the BLAST search, and formed an individual clade in the phylogenetic tree (Figure 2). These two species were distinctly evolved from *Calostoma* mushrooms in China, Malaysia, Japan, Australia, and the USA.

### 3.3. Optimal Medium for Mycelial Pellet Cultivation

**Figure 3:**
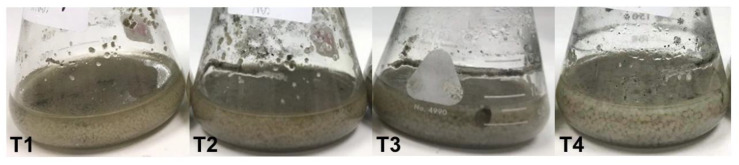
Mycelial pellet formation during 5-day cultivation in T1–T4 media.

The optimal medium for fungal mycelial pellet growth was determined to promote species conservation. A statistically significant difference (*p* ≤ 0.05) was observed in mycelial pellet biomass in the four tested growth media (Figure 3). The T3 medium (potato + sucrose + peptone) provided the highest mycelial pellet fresh weight at 23.12 ± 2.01 g FW/100 mL, followed by T2 (potato + sucrose) at 16.57 ± 1.28 g FW/100 mL, T1 (PDB) at 13.25 ± 1.50 g FW/100 mL, and T4 (potato + sucrose + malt extract) at 12.60 ± 0.71 g FW/100 mL (Table 1).

The dry weight of the mycelial pellet followed the similar trend (Table 1). The lowest pH value of 3.64 was recorded in T1 at day 5, whilst the highest pH of 4.70 was found in T3 (Table 1).

### 3.4. FTIR Spectra

**Figure 4:**
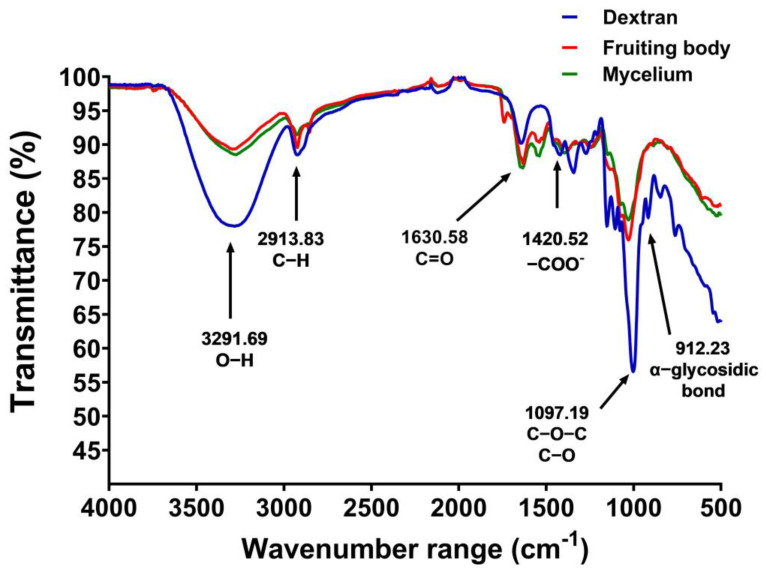
FTIR spectra of the gelatinous fruiting body (red), mycelial pellet extract (green), and dextran (blue).

FTIR spectroscopy was used to analyze the polysaccharide structures. The FTIR spectra of the dried gelatinous fruiting body and mycelial pellet extract both exhibited covalent bond molecular vibrations (4000–500 cm^−1^). The infrared absorption characteristics of the polysaccharides were observed at frequencies of 3291.69 cm^−1^ (O−H), 2913.83 cm^−1^ (C−H), 1630.58 cm^−1^ (C=O), 1420.52 cm^−1^ (−COO^-^), 1097.19 cm^−1^ (C−O−C, C−O), and 912.23 cm^−1^ (α-glycosidic bonds) (Figure 4), with dextran standard used for comparison.

### 3.5. Antioxidant, α-Glucosidase Inhibition, and Antimicrobial Activities

The antioxidant, α-glucosidase inhibition, and antimicrobial activities of the gelatinous fruiting body extract exhibited significantly higher % DPPH inhibition of 57.96 ± 1.26%, DPPH scavenging activity of 2.85 ± 0.02 mg Trolox/g, FRAP value of 1.73 ± 0.01 mg FeSO_4_/g, TPC and TFC values of 1.00 ± 0.05 mg GAE/g and 3.51 ± 0.18 mg RE/g, and α-glucosidase inhibition of 73.18 ± 5.24% than the mycelial pellet extracts grown in the four different media (Table 2). The T3 medium provided the highest bioactivities among the four media. Hence, the mycelial pellet extract in the T3 medium was chosen to test for antimicrobial activity against three pathogenic bacteria (Gram-positive *S. agalactiae* and *S. aureus* and Gram-negative *E. coli*) using the agar disc diffusion method and compared with the gelatinous fruiting body extract and penicillin (as a positive control).

**Figure 5:**
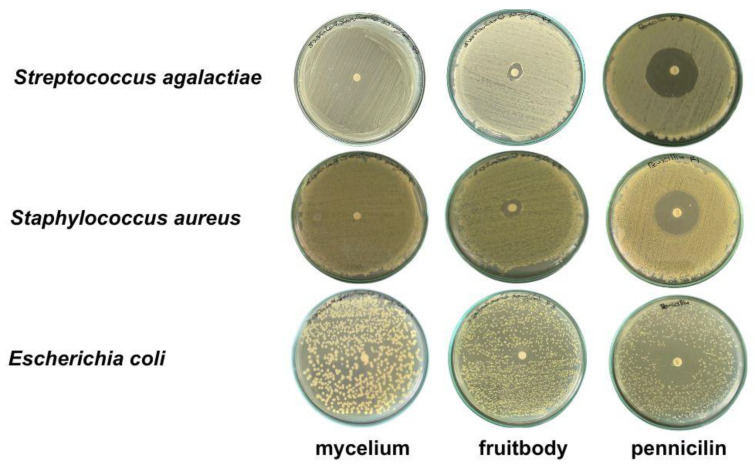
Antimicrobial activity against three pathogenic bacteria using agar disc diffusion method.

The gelatinous fruiting body extract exhibited antimicrobial activity against all three pathogens in descending order of *S. aureus, E. coli*, and *S. agalactiae* (Table 3 and Figure 5), while the mycelial pellet extract in the T3 medium did not inhibit the pathogens.

### 3.6. Cytotoxicity against Cancer Cells

**Figure 6:**
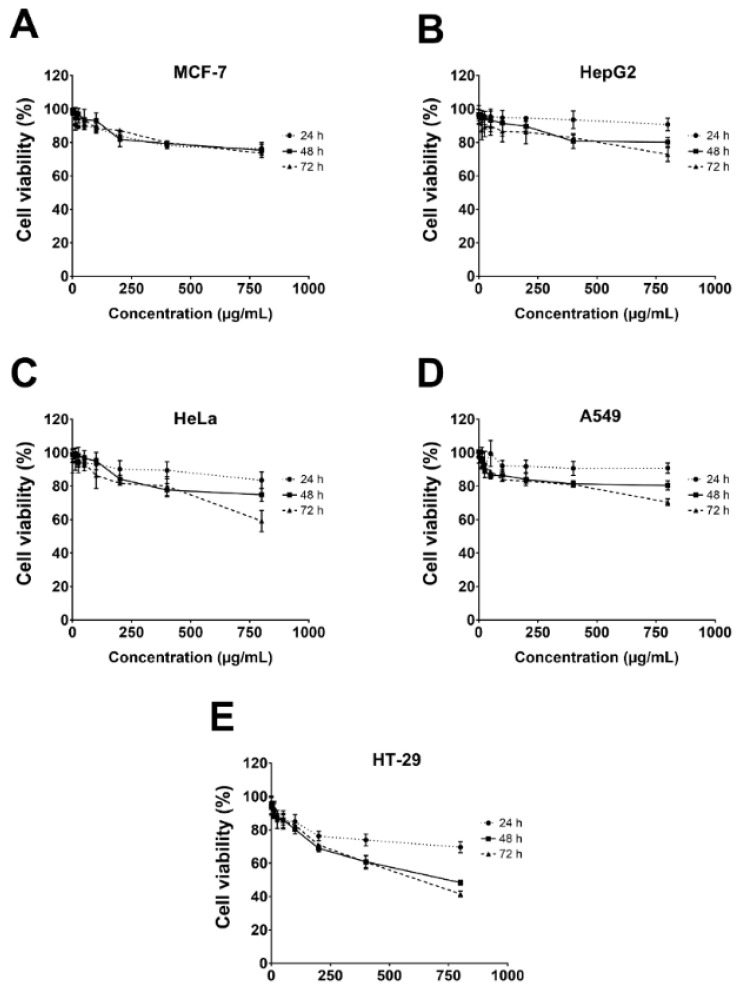
Cell viability of five cancer cells after gelatinous fruiting body extract treatment for 72 h.

The cytotoxicity of the gelatinous fruiting body extract towards the five cancer cells, i.e., HepG2 (liver), MCF-7 (breast), HeLa (cervical), A549 (lung), and HT-29 (colon), was conducted using the MTT assay. Results showed that cytotoxicity was only weakly effective towards HT-29 at very high concentrations (IC_50_ of 770.6 ± 5.0 µg/mL at 72 h and %cell viability of 41.6 ± 1.8% at 800 µg/mL at 72 h) (Figure 6).

### 3.7. ROS Generation and Apoptosis Induction in HT-29 Cells

**Figure 7:**
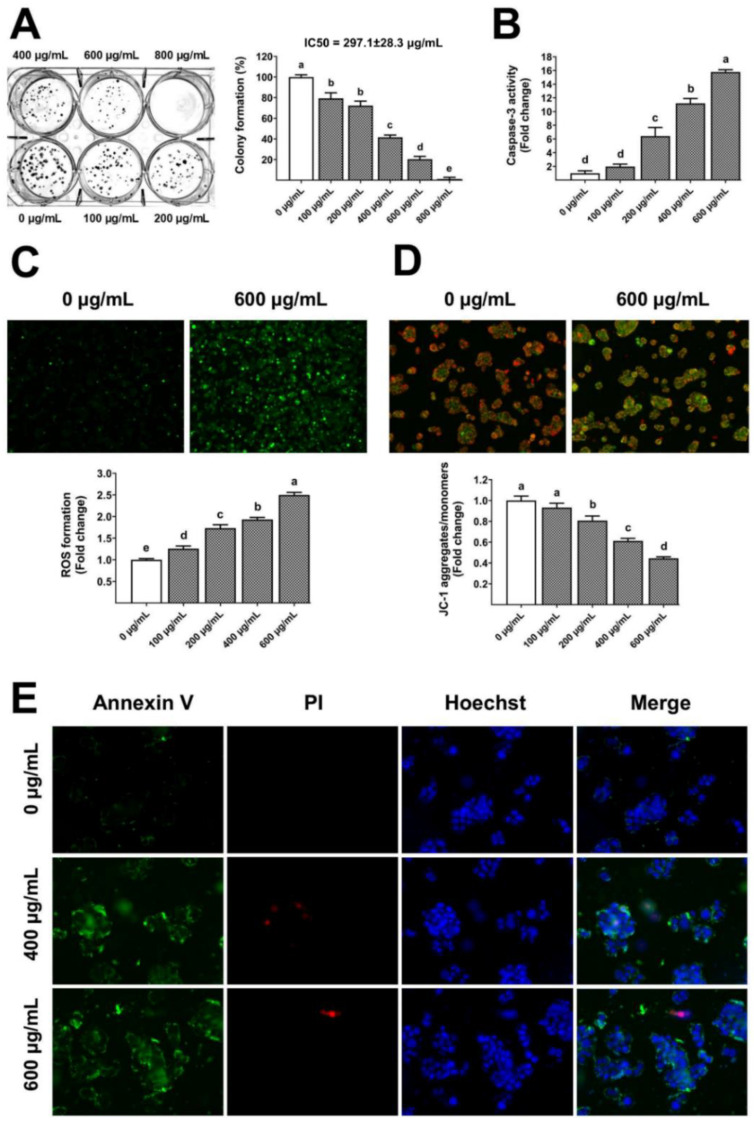
Apoptosis induction by the gelatinous fruiting body extract: (**A**) anti-colony formation, (**B**) caspase-3 activity, (**C**) ROS formation, (**D**) mitochondrial transmembrane potential, and (**E**) annexin V/PI staining for apoptotic stage test. Different lowercase letters on the bars indicate significant differences (*p* < 0.05).

The gelatinous fruiting body extract also showed long-term antiproliferative capacity (IC_50_ 297.1 µg/mL) towards HT-29 (Figure 7A), leading to a dose-dependent increase in caspase-3 activity (Figure 7B). Traditionally, ROS from mitochondria and other cellular sources have been considered harmful byproducts of metabolism [33], whereas ROS have been reported to induce apoptosis [34]. In this study, the groups treated with gelatinous fruiting body extract exhibited notably elevated levels of ROS compared to the control group. The gelatinous fruiting body extract induced a dose-dependent accumulation of ROS in HT-29 cells, as seen by higher green fluorescence (Figure 7C), and also induced changes in mitochondrial transmembrane potential towards apoptosis. The untreated control cells showed red fluorescence of JC-1 dye aggregates, indicating active mitochondria, but the tested extract showed a dose-dependent enhancement in green fluorescence of free JC-1 dye monomers, indicating a decreased mitochondrial activity (Figure 7D). Hoechst, Annexin V-FITC, and PI triple fluorescence pictures revealed HT-29 cell apoptosis after 400 and 600 µg/mL extract treatments. Blue signals showed the cell nucleus, green signals showed Annexin V, and red signals showed PI. The untreated control cells had fewer Annexin V-FITC and PI signals, whereas the gelatinous fruiting body extract treatment caused significant green and weak red fluorescence densities, indicating early apoptosis (Figure 7E).

**Figure 8:**
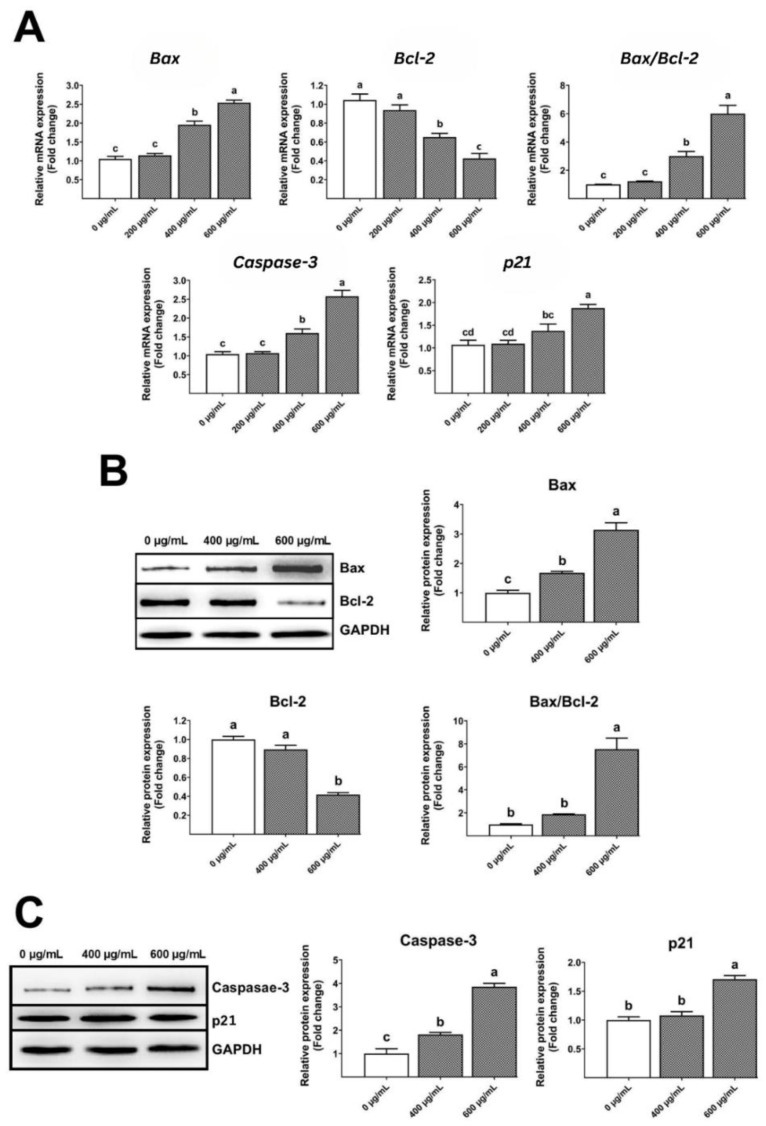
Effects of the gelatinous fruiting body extract on apoptosis gene and protein expressions: (**A**) real-time analysis of gene expressions, (**B**) Western blot analysis of Bax and Bcl-2 protein expressions, and (**C**) Western blot analysis of caspase-3 and p21 protein expressions. Different lowercase letters on the bars indicate significant differences (*p* < 0.05).

Real-time PCR research showed that the gelatinous fruiting body extracts increased *Bax*, *Bax/Bcl-2 ratio, caspase-3*, and *p21* levels but decreased *Bcl-2* levels (Figure 8A) in a dose-dependent manner, leading to apoptosis and cell cycle arrest. Similarly, Western blot examination showed that the gelatinous fruiting body extracts enhanced Bax and Bax/Bcl-2 ratio protein expressions and decreased Bcl-2 levels in a dose-dependent manner (Figure 8B). Similar trends were seen in caspase-3 and p21 protein expressions (Figure 8C).

## 4. Discussion

The investigation of gasterocarp macro- and micromorphology using scanning electron microscopy of the spores and capillitium and the phylogenetic analysis of *C. insigne* improved the understanding of this species. The structure of basidiospores at a microscopic level demonstrated the significance of functional morphology in relation to their ability to reproduce. The star-shaped formation served as a protective barrier for the basidiospore, shielding it from adverse conditions, aiding in its attachment to the substrate, and enabling its survival in a less than ideal environment [29]. Deloya-Olvera et al. (2023) [35] found that *C. insigne* differed from *C. lutescens*, *C. ravenelii*, and *C. cinnabarinum* in macro- and micromorphology.

Molecular comparison of sequences in GenBank showed 99% similarity with the most similar species, *C. insignis* Arora98-31 (EU718092.1) from Thailand [32]. The clade topology differed from other *Calostoma* species from other countries. Both our phylogenetic study and a previous report showed that *C. insigne* from Thailand evolved differently from other species in the same genus [35]. Compared to other Boletales, *Calostoma* sp. has a large number of estimated nucleotide mutations [36], indicating exceptional morphological divergence [37]. Variations in the environment complicate identification. The humid conditions of deciduous woodlands generate a denser and more gelatinous exoperidium in *Calostoma* species [38].

This is the first scientific report of the edible and rare *Calostoma* mushroom with bioactive potential including antioxidant, antimicrobial, anti-glucosidase, and anticancer properties. Due to the lack of previous reports on the bioactivities of *Calostoma* species, certain mushrooms in the family Sclerodermataceae, primarily including earthball mushrooms, were compared with our puffball mushroom; however, not all the mentioned bioactivities were documented for earthball mushrooms. The melanin extract from *Scleroderma citrinum* mushroom was found to exhibit promising antioxidant and antibacterial properties [39]. The TPC value for crude melanin extract of *S. citrinum* was 0.18 ± 0.01 mg GAE/g at 1 mg/mL, which was significantly lower than our study (TPC value 1.00 ± 0.05 mg GAE/g at 20 mg/mL). The antioxidant activity of *S. citrinum* crude melanin extract using 2,2’-azino-bis (3-ethylbenzthiazoline-6-sulfonic acid) (ABTS) assay was 75.66 ± 0.13% at 1 mg/mL compared to *C. insigne* with DPPH inhibition of 57.96 ± 1.26% at 20 mg/mL. The *S. citrinum* melanin extract showed antibacterial activity against *Pseudomonas aeruginosa* and *Enterococcus faecalis* but no antibacterial activity towards *Bacillus cereus*, *E. coli*, and *S. aureus* [39]. In contrast, our results showed the antimicrobial potential of *C. insigne* polysaccharide against *E. coli* and *S. aureus*. These significant differences in TPC values, antioxidant activities, and antimicrobial activity between the *S. citrinum* melanin extract and our *C. insigne* polysaccharide extract may result from different bioactive molecules present in the tested extracts and also different extract concentrations used in the assays. *Pisolithus arhizus*, an ectomycorrhizal fungus known as an earthball, also demonstrated that its two triterpenoid extracts caused a modest dose-dependent decrease in cell viability in U87MG glioma and Jurkat leukemia cell lines [40]. Both the fungal methanol and ethanol extracts possessed antioxidant and antibacterial properties, and they also exhibited significant cytotoxicity against HT-29 colon cancer cells, with impact directly proportional to the concentration of the extracts [41]. These findings concurred with our results showing that *C. insigne* fruiting body extract exerted antioxidant, antibacterial, and cytotoxic properties towards HT-29 cells.

The gelatinous fruiting body had a polysaccharide structure, as shown by the FTIR output. Our results indicated that *C. insigne* demonstrated a good antioxidant ability by scavenging free radicals, with a higher DPPH scavenging activity of 57.96% than oyster mushroom (*Pleurotus florida*) and milky mushroom (*Calocybe indica*) (0.2–1.0 mg/mL) at 37.04 and 28.04%, respectively [42], when used at a higher concentration (20 mg/mL). The *C. insigne* gelatinous fruiting body also showed antibacterial action against Gram-positive *S. agalactiae* (causing fish infection), Gram-positive *S. aureus*, and Gram-negative *E. coli* (causing human gut disorders). These results suggested that the gelatinous fruiting body polysaccharide may have potential applications in both aquaculture and human gut health. Basidiomycetes are antibacterial because they contain physiologically active compounds such as polysaccharides [43].

*C. insigne* also inhibited α-glucosidase enzyme activity by 73.18% at 20 mg/mL, suggesting that by blocking glucosidase, mushroom polysaccharides may be used to treat diabetes by reducing postprandial hyperglycemia and other diseases [44]. Previous research found that oyster mushroom polysaccharide inhibited α-glucosidase [45]. Polysaccharide biological activities may be linked to their structure or conformation [46], which contribute to their ability to bind amino acids, peptides, proteins, polyphenols, steroids, and other substances.

The gelatinous fruiting body extract of *C. insigne* was significantly more bioactive than the laboratory-grown mycelial pellet extract, despite the similar functional groups and chemical bond characteristics in polysaccharides based on the FTIR analysis. Nuclear Magnetic Resonance (NMR) spectroscopy analysis may be required to determine more detailed molecular structural and dynamic information. The differences in bioactivities between both extracts may lie in the diverse chemical and physical surroundings for production of the gelatinous fruiting body and the laboratory-grown mycelial pellet (natural forest habitats vs. laboratory flasks). Both fungal cells were subjected to varying environmental/laboratory conditions of relative humidity, oxygen levels, light exposure, temperature fluctuations, shear stress, nutrition availability, and symbiotic microbial community (in case of naturally found fruiting bodies) [47]. These may lead to differential growth rates, developmental stages, gene expressions, enzyme activities [47,48], defense compounds [49], volatiles [50], minerals, antioxidants [51], and different sugar monomers composing the polysaccharides [52], contributing to bioactivity differences between the extracts. Further investigations are required to determine the optimal cultivation of mycelia to confer similar health-beneficial bioactivities as found in the fruiting bodies in natural forest habitats.

Focus must be on preserving the fungal specimen and producing fungal biomass. Here, the T3 medium (potato + sucrose + peptone) was found to be optimal for fungal mycelial biomass production, with the highest bioactivities, probably because peptone in the T3 medium is a rich source of amino acids, vitamins, and minerals, which are necessary for the synthesis of proteins/enzymes, bioactive compounds, and other cellular components. In contrast, sucrose in the T2 medium and malt extract in the T4 medium serve as sources of carbohydrates, which can be easily utilized for fermentation but were probably not sufficient for high biomass production or bioactive compound syntheses. 

The *C. insigne* gelatinous fruiting body ethanolic extract showed a dose-dependent suppression of HT-29 cell growth (IC_50_ 770.6 µg/mL at 72 h), indicating very weak cytotoxic action. Cell viability was reduced by up to 58% after 72 h of treatment at 800 µg/mL concentration. The clonogenic assay revealed a greater antiproliferative capacity (IC_50_ 297.1 µg/mL) for 14 days, suggesting that longer-term therapy was more effective. The molecular mechanisms of gelatinous fruiting body extract-induced HT-29 cell cycle arrest and apoptosis were examined. Chemo-preventive medicines stall the cell cycle and produce apoptosis by increasing ROS and disturbing redox equilibrium [53,54]. Then, ROS accumulation causes ER stress, DNA damage, and downstream protein expression alterations such as p53, which activate or repress cell cycle arrest and death genes to modulate cell stress response [55]. DNA synthesis is stopped by p21, a cell cycle checkpoint gene regulated by p53 [56]. The gelatinous fruiting body extracts enhanced ROS and p21 in a dose-dependent manner, suggesting that it may affect cell cycle regulatory components required to block the cell cycle.

**Figure 9:**
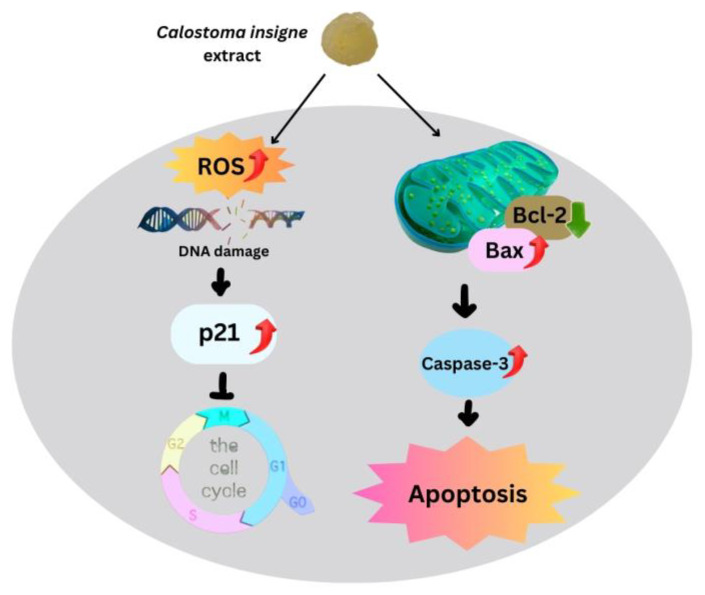
Proposed apoptotic mechanisms of the puffball mushroom (*C. insigne*) gelatinous fruiting body extract in HT-29 cells.

Intrinsic mitochondrial apoptosis is the main cancer treatment [57,58,59]. ROS and antioxidant imbalances kill cells [60]. Increased mitochondrial ROS buildup enhances outer mitochondrial membrane permeability, accelerating cytochrome c transport from intermembrane to cytosol [61], with *Bcl-2* or *Bcl-xL* expression controlling this mechanism [62,63]. Here, the gelatinous fruiting body extract increased *Bax* expression but decreased *Bcl-2*, resulting in a higher *Bax/Bcl-2* ratio than in the control group, which accelerated cytochrome c transport into the cytoplasm. Cytochrome c stimulates initiator and effector caspases via procaspase-9. The gelatinous fruiting body extracts increased caspase-3 in HT-29 in a dose-dependent manner, causing apoptosis. Figure 9 shows how the gelatinous fruiting body extract arrested the cell cycle and induced apoptosis in HT-29 cells.

## 5. Conclusions

This is the first report detailing the bioactivities of the rare edible mushroom *C. insigne*. The crude ethanolic gelatinous fruiting body extracts containing polysaccharide displayed antioxidant activity against DPPH, reducing power, antimicrobial activity against the bacterial pathogens, *S. aureus*, *E. coli*, and *S. agalactiae,* in the gut and in aquaculture, and glucosidase inhibition for diabetic treatment and anticancer effects towards the colon cancer cell line HT-29 via an intrinsic apoptotic pathway. Further research on the in vivo preventative effects of *C. insigne* extracts and the molecular structure of the active chemicals is still required. The optimal medium for laboratory-grown mycelial pellets was determined for the production of the fungal biomass; however, this did not result in similar bioactivities compared to the gelatinous fruiting body, which showed significantly higher bioactivities. Global warming is now a serious threat to the extinction of this fungal species, and cultivating laboratory-grown mycelia offers a more effective means of species propagation as the preferred approach for polysaccharide production. However, further investigations are necessary.

## Figures and Tables

**Table 1:** Mycelial pellet biomass and pH during 5-day cultivation in T1–T4 media.

Media	Mycelial Fresh Weight (FW) (g/100 mL)	Mycelial Dry Weight (DW) (g/100 mL)	pH Day 0	pH Day 5
T1: PDB	13.25 ± 1.50 ^c^	1.28 ± 0.16 ^d^	5.00 ± 0.00	3.64 ± 0.03 ^c^
T2: potato + sucrose	16.57 ± 1.28 ^b^	2.17 ± 0.21 ^b^	5.00 ± 0.00	4.31 ± 0.02 ^b^
T3: potato + sucrose + peptone	23.12 ± 2.01 ^a^	2.74 ± 0.09 ^a^	5.00 ± 0.00	4.70 ± 0.03 ^a^
T4: potato + sucrose + malt extract	12.60 ± 0.71 ^c^	1.88 ± 0.11 ^c^	5.00 ± 0.00	4.37 ± 0.05 ^b^

Different letters in columns indicate statistical differences (*p* ≤ 0.05).

**Table 2:** Bioactivities of the gelatinous fruiting body and mycelial pellet extracts.

Extract	% DPPH Inhibition	DPPH Scavenging Activity (mg Trolox/g)	FRAP Value (mg FeSO_4_/g)	TPC (mg GAE/g)	TFC (mg RE/g)	% α-Glucosidase Inhibition
Fruiting body	57.96 ± 1.26 ^a^	2.85 ± 0.02 ^a^	1.73 ± 0.01 ^a^	1.00 ± 0.05 ^a^	3.51 ± 0.18 ^a^	73.18 ± 5.24 ^a^
Mycelium T1	37.90 ± 0.25 ^d^	1.26 ± 0.02 ^d^	0.75 ± 0.02 ^e^	0.39 ± 0.04 ^d^	1.22 ± 0.20 ^d^	ND
Mycelium T2	36.72 ± 0.65 ^d^	1.17 ± 0.05 ^e^	0.95 ± 0.01 ^c^	0.49 ± 0.04 ^c^	1.42 ± 0.06 ^cd^	ND
Mycelium T3	44.60 ± 0.48 ^b^	1.76 ± 0.04 ^b^	1.14 ± 0.01 ^b^	0.61 ± 0.01 ^b^	1.72 ± 0.10 ^b^	49.88 ± 4.91 ^b^
Mycelium T4	42.59 ± 0.36 ^c^	1.61 ± 0.03 ^c^	0.82 ± 0.02 ^d^	0.48 ± 0.03 ^c^	1.49 ± 0.10 ^bc^	ND

Different letters in the columns indicate statistical differences (*p* ≤ 0.05), ND = not determined, FRAP = ferric ion-reducing antioxidant power, TPC = total phenolic content, and TFC = total flavonoid content.

**Table 3:** Antimicrobial activity against pathogenic bacteria using agar disc diffusion method.

Samples	Inhibition Zone (mm Diameter)		
	*S. aureus*	*S. agalactiae*	*E. coli*
Mycelium T3 (20 mg/mL)	ND	ND	ND
Fruiting body (20 mg/mL)	15.00 ± 1.00 ^bA^	12.67 ± 0.29 ^bB^	13.50 ± 0.50 ^bA^
Penicillin (10 µg/mL)	59.27 ± 0.91 ^aA^	61.00 ± 1.00 ^aA^	54.00 ± 1.00 ^aB^

Different lowercase and uppercase letters in the same columns and rows indicate significant differences (*p* < 0.05). ND = not detected.

## Data Availability

The data used to support the findings of this study are available from the corresponding author upon request.

## Supplementary Materials

The following supporting information can be downloaded at: https://www.mdpi.com/article/10.3390/foods13010113/s1. Table S1: Primers for gene analysis.
