# Supplementary material for: Exploring the Bioactive Potential of Calostoma insigne, an Endangered Culinary Puffball Mushroom, from Northeastern Thailand
Source: Foods. 2023 Dec 28;13(1):113. doi: 10.3390/foods13010113 (PMC10778563; doi:10.3390/foods13010113)
Supplement: Supplementary file 1 [file foods-13-00113-s001.zip › foods-2779821-supplementary.pdf]

Table S1 Primers for gene analysis

Gene	Primer	Primer Sequence (5’-3’)	Size (bp)
GAPDH	Forward	CACTGCCAACGTGTCAGTGGTG	121
	Reverse	GTAGCCCAGGATGCCCTTGAG	
BAX	Forward	TGCTTCAGGGTTTCATCCAG	170
	Reverse	GGCGGCAATCATCCTCTG	
BCL2	Forward	AGGAAGTGAACATTTCGGTGAC	149
	Reverse	GCTCAGTTCCAGGACCAGGC	
Caspase-3	Forward	GGCGCTCTGGTTTTCGTTAAT	121
	Reverse	TCCAGAGTCCATTGATTCGCT	
p21	Forward	AGTCAGTTCCTTGTGGAGCC	109
	Reverse	GCATGGGTTCTGACGGACAT	
